# Vesiclepedia 2024: an extracellular vesicles and extracellular particles repository

**DOI:** 10.1093/nar/gkad1007

**Published:** 2023-11-11

**Authors:** Sai V Chitti, Sriram Gummadi, Taeyoung Kang, Sanjay Shahi, Akbar L Marzan, Christina Nedeva, Rahul Sanwlani, Kyle Bramich, Sarah Stewart, Monika Petrovska, Biswadeep Sen, Alper Ozkan, Maria Akinfenwa, Pamali Fonseka, Suresh Mathivanan

**Affiliations:** Department of Biochemistry, La Trobe Institute for Molecular Science, La Trobe University, Melbourne, Victoria 3086, Australia; Department of Biochemistry, La Trobe Institute for Molecular Science, La Trobe University, Melbourne, Victoria 3086, Australia; Department of Biochemistry, La Trobe Institute for Molecular Science, La Trobe University, Melbourne, Victoria 3086, Australia; Department of Biochemistry, La Trobe Institute for Molecular Science, La Trobe University, Melbourne, Victoria 3086, Australia; Department of Biochemistry, La Trobe Institute for Molecular Science, La Trobe University, Melbourne, Victoria 3086, Australia; Department of Biochemistry, La Trobe Institute for Molecular Science, La Trobe University, Melbourne, Victoria 3086, Australia; Department of Biochemistry, La Trobe Institute for Molecular Science, La Trobe University, Melbourne, Victoria 3086, Australia; Department of Biochemistry, La Trobe Institute for Molecular Science, La Trobe University, Melbourne, Victoria 3086, Australia; Department of Biochemistry, La Trobe Institute for Molecular Science, La Trobe University, Melbourne, Victoria 3086, Australia; Department of Biochemistry, La Trobe Institute for Molecular Science, La Trobe University, Melbourne, Victoria 3086, Australia; Department of Biochemistry, La Trobe Institute for Molecular Science, La Trobe University, Melbourne, Victoria 3086, Australia; Department of Biochemistry, La Trobe Institute for Molecular Science, La Trobe University, Melbourne, Victoria 3086, Australia; Department of Biochemistry, La Trobe Institute for Molecular Science, La Trobe University, Melbourne, Victoria 3086, Australia; Department of Biochemistry, La Trobe Institute for Molecular Science, La Trobe University, Melbourne, Victoria 3086, Australia; Department of Biochemistry, La Trobe Institute for Molecular Science, La Trobe University, Melbourne, Victoria 3086, Australia

## Abstract

Vesiclepedia (http://www.microvesicles.org) is a free web-based compendium of DNA, RNA, proteins, lipids and metabolites that are detected or associated with extracellular vesicles (EVs) and extracellular particles (EPs). EVs are membranous vesicles that are secreted ubiquitously by cells from all domains of life from archaea to eukaryotes. In addition to EVs, it was reported recently that EPs like exomeres and supermeres are secreted by some mammalian cells. Both EVs and EPs contain proteins, nucleic acids, lipids and metabolites and has been proposed to be implicated in several key biological functions. Vesiclepedia catalogues proteins, DNA, RNA, lipids and metabolites from both published and unpublished studies. Currently, Vesiclepedia contains data obtained from 3533 EV studies, 50 550 RNA entries, 566 911 protein entries, 3839 lipid entries, 192 metabolite and 167 DNA entries. Quantitative data for 62 822 entries from 47 EV studies is available in Vesiclepedia. The datasets available in Vesiclepedia can be downloaded as tab-delimited files or accessible through the FunRich-based Vesiclepedia plugin.

## Graphical Abstract

**Graphical Abstract:**
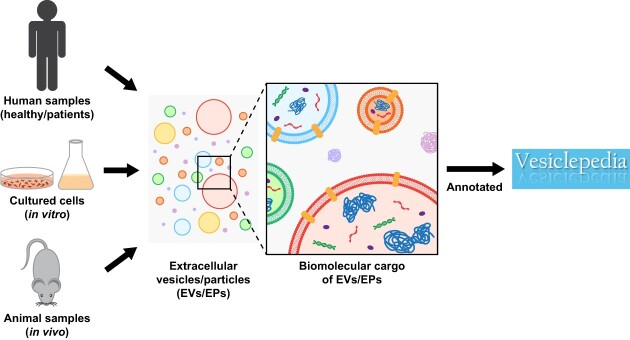

## Introduction

**Figure 1.**
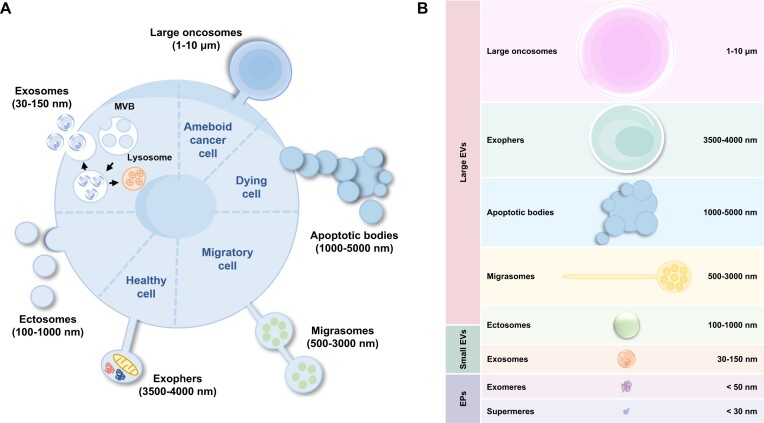
Subtypes of EVs based on their biogenesis pathways. (**A**) Based on their biogenesis pathways, EVs are classified into various subtypes. Exosomes, range from 30 to 150 nm, originate from the endosomal pathway. Microvesicles/ectosomes, range from 100 to 1000 nm, are produced through outward budding of the plasma membrane. Migrasomes are the vesicles that are left behind by the migratory cells and are in the size of 500 to 3000 nm. Apoptotic bodies with a diameter up to 1–5 μm are the largest class of EV subtypes and are produced through the blebbing and protrusions of the apoptotic cell membrane during cell death. Large oncosomes, range from 1 to 10 μm, are the large class of cancer-derived EVs originating from the shedding of membrane blebs. (**B**) Based on the operational terms and size, EVs and particles are broadly classified as large EVs, small EVs and EPs.

Extracellular vesicles (EVs) are nanosized membrane bound vesicles that are shed by cells into extracellular space (1). They are known to be secreted ubiquitously by cells from all domains of life from archaea to eukaryotes (2). In addition to EVs, some of the mammalian cells are also reported to secrete extracellular particles (EPs) (3–5). Over the past two decades, EVs have been proposed to have a key role in multiple pathophysiological processes due to their signalling capabilities (6–9). Furthermore, these signalling moieties are now known to not only function within bounds of an individual by mediating signalling between distant cells but also have a much more complex role in mediating cross-species and inter-kingdom communication (10–12). EVs have been classified into various subtypes which differ based on their biogenesis and origin. Further, these EV subtypes also differ in their size, cargo components and consequently their functions (13) (Figure 1). Exosomes (30–150 nm) are endocytic vesicles as they originate due to the fusion of multivesicular bodies with the plasma membrane (14). On the contrary, ectosomes (100–1000 nm) originate due to budding of plasma membrane (13,15). While live cells participate in active secretion of exosomes and ectosomes, apoptotic or dying cells have been long known to secrete apoptotic bodies (50–5000 nm). Similar to ectosomes, apoptotic bodies also originate due to budding of plasma membrane. Migrasomes (500–3000 nm) are shed by migrating cells while large oncosomes (1000–10 000 nm) are shed by amoeboid cancer cells (16,17). In EPs, exomeres and supermeres are smaller than 50 nm in size and their mode of biogenesis remains unknown (3).

In terms of their molecular composition, EVs sequester a diverse and rich cargo of proteins, lipids, and nucleic acids, aiding in their safe delivery from the donor to recipient cells (1). As the function of the EVs is context dependent, the ensuing cargo is altered according to the pathophysiological conditions of the host cells that secrete them. Hence, a catalogue of the EV cargo (nucleic acids, proteins, metabolites and lipids) in various conditions could aid in identifying an EV fingerprint that are specific to a tissue, cell type, and/or pathology. Here, we describe an update of Vesiclepedia (http://www.microvesicles.org) which is an online database that contains a list of RNA, proteins, lipids and metabolites that are identified in EVs and EPs. Furthermore, Vesiclepedia also contains EV and EP-associated DNA data that were obtained from 52 studies including 167 data entries for oncogenes and mutated genes.

## Vesiclepedia

**Figure 2.**
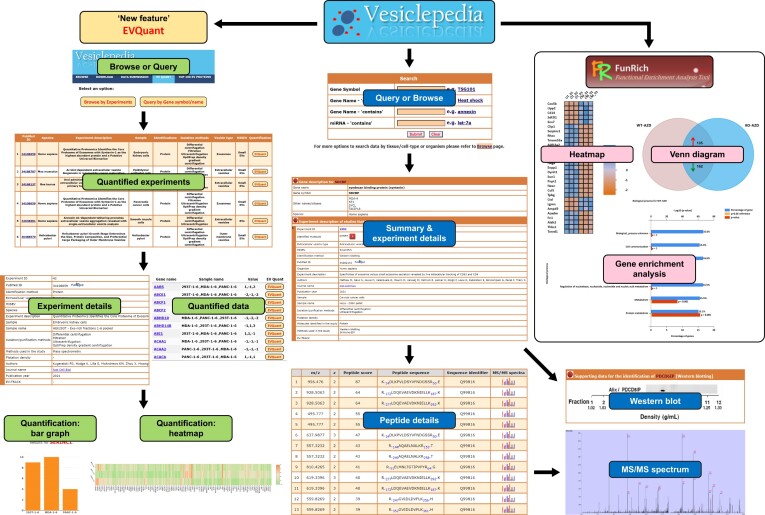
Snapshot of Vesiclepedia pages with the example of EV enriched protein TSG101. Search for the molecule of interest provides gene summary page that contains the data on the molecule, experimental details pertaining to the studies that identified the molecule, Western blotting data to support the identification (if applicable) and mass spectrometry-based peptide details along with the tandem MS spectra is provided wherever possible. EV-QUANT provides quantitative data for EV studies. For the experiment of interest, the quantification for the cargo identified is available as data table and abundant cargo are visualised as heatmap. Alongside that, for the gene of interest in the study, the relative quantification can be visualised by bar graph. Vesiclepedia plugin in FunRich can be used to automatically download data for downstream analysis.

Vesiclepedia is built using a combination of MySQL for the background database, Zope content management system and Python as the programming language that connects the database to the content management system. The website allows the user to query or browse the database as well as download the entire data as tab delimited files freely. Upon querying for a gene or browsing through EV studies, users can get additional information on the gene summary, EV isolation method used, flotation density (if provided), EV subtype and sample source (Figure 2). The cargo data catalogued in Vesiclepedia is obtained either through community annotation wherein the authors submit their datasets or through manual curation of the literature. While exosomes or small EV specific databases such as ExoCarta (18) exists, Vesiclepedia (http://www.microvesicles.org) contains cargo from EV subtypes and EPs (19).

## New features and data addition

### Incorporation of additional EV subtypes and EPs

**Figure 3.**
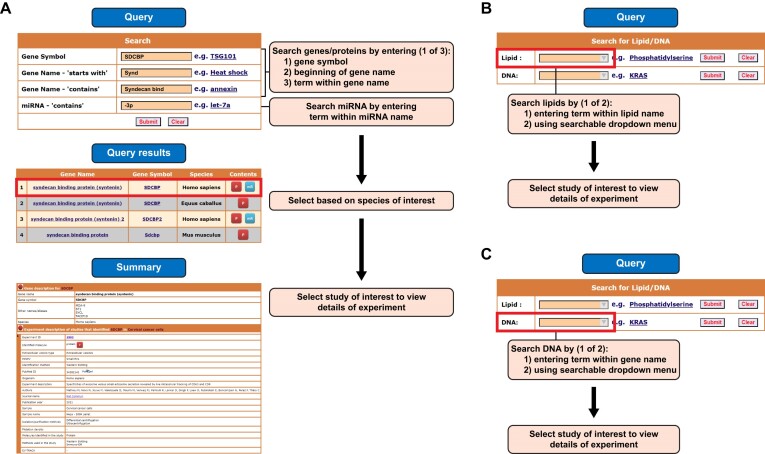
Schematic workflow of Vesiclepedia query page. Multiple query options are available for users to access EV/EP contents (**A**) Search for the proteins/miRNA by entering either gene symbol, gene name or miRNA name to retrieve all the related experiment details. (**B**) Search for the lipid of interest by entering the lipid name in searchable dropdown menu to retrieve experimental details. (**C**) For DNA, use searchable dropdown option to retrieve experimental details.

In addition to EV subtypes like exosomes, ectosomes and apoptotic bodies, new studies that profiled the cargo of migrasomes are added to Vesiclepedia. Similarly, for the first time, recently described EPs including exomeres and supermeres cargo are annotated and catalogued in Vesiclepedia. It has to be noted that the biogenesis pathway of exomeres and supermeres are unknown and hence the availability of nucleic acids/protein/lipid catalogue would spur more studies aimed at understanding the biogenesis pathways (Figure 3).

## EV-QUANT

Previously, Vesiclepedia data was annotated as present in a specific study and EV subtype while the quantification data was non-existent. In the current update, a new feature EV-QUANT is added where it allows for relative quantification between EV protein/RNA/lipid cargo samples within one specific study. Currently, 47 studies encompassing 62 822 entries have quantitative information in Vesiclepedia. As every study had different experimental procedures and sample processing, quantitative cross comparison between studies is currently not possible. However, with the availability of new high throughput datasets, robust uniform pipelines can be developed in the future and cross comparison between studies is possible if the experimental procedures are identical.

## MISEV, FunRich and EV-TRACK

The EV subtype annotated within Vesiclepedia is now updated according to the MISEV guidelines and categorised based on operational terms such as EVs, small EVs, large EVs and EPs. This allows for the further standardization of the EV subtype nomenclature. Furthermore, author described terminology of the EV subtype is also added for users who may be benefited.

In addition, Vesiclepedia data can be downloaded into FunRich (20,21) software and used for various functional enrichment analysis. Most importantly, the downloaded data can be filtered based on several parameters including sample source, cell type, isolation method, species, and EV subtype. When it relates to EVs, The EV-TRACK consortium reports an EV-METRIC based on various EV isolation and characterisation parameters that are reported in a particular study. Each study annotated in EV-TRACK receives a score that measures the transparency of reporting experimental meta-annotations in publications (22). The score is now added to Vesiclepedia and linked to both published and unpublished studies.

## Data update

Vesiclepedia currently contains 3533 EV studies which is more than 2-fold increase in the number of EV studies catalogued in the database since the last 2019 update. Furthermore, Vesiclepedia has incorporated data from 56 6911 protein entries, 50 550 RNA entries, 3839 lipid, 192 metabolite and 167 DNA entries (Table 1). Quantitative data is available for 62 822 entries annotated from 47 studies currently in Vesiclepedia. Overall, a total of 252 sample sources from 56 organisms are currently catalogued in Vesiclepedia.

**Table 1.** Statistics of data in Vesiclepedia

1	EV studies	3533
2	DNA entries	167
3	RNA entries	50 550
4	mRNA entries	27 692
5	Unique mRNA	17 008
6	miRNA entries	22 858
7	Unique miRNA	5253
8	Protein entries	566 911
9	Unique proteins	46 687
10	Lipid entries	3839
11	Metabolite entries	192
12	Organisms	56
13	Sample sources	252
14	Quantification entries	62 822

## Limitations of data available in Vesiclepedia

As Vesiclepedia contains curated or author submitted data from published literature, the quality of the data correlates with the EV study of interest. It is well established that the field of EVs lack stringent nomenclature and the EV isolation protocol differs between the studies and laboratories. EV/EP isolation method critically impacts the purity of the isolated sample, type of EVs/EPs and the cargo that are associated with EVs. Hence, we emphasise caution while using the data from Vesiclepedia for any downstream analysis. Users may need to pay attention to the isolation methods used and the meta-annotations pertaining to the experiment to filter specific high-quality datasets of interest. Similarly, all the top 100 proteins often identified in EV studies cannot be termed as EV markers or enriched proteins.

## Data Availability

Vesiclepedia is a free (academic and commercial) web-based compendium of EVs and EPs that is available at http://www.microvesicles.org. The data present in Vesiclepedia is available freely as tab-delimited files through the download page or through FunRich enrichment analysis tool (http://www.funrich.org).
